# Students’ perception of e-learning during the Covid-19 pandemic: a survey study of Iranian nutrition science students

**DOI:** 10.1186/s12909-023-04585-7

**Published:** 2023-08-22

**Authors:** Ghazaleh Eslamian, Mehrnoosh Khoshnoodifar, Shirin Malek

**Affiliations:** 1 Department of Cellular and Molecular Nutrition, Faculty of Nutrition and Food Technology, National Nutrition and Food Technology Research Institute, Shahid Beheshti University of Medical Sciences, Tehran, Iran; 2 Department of e-learning, Virtual School of Medical Education and Management, Shahid Beheshti University of Medical Sciences, NO. 2823, Valiasr St, P.O.Box: 1966645641, Tehran, Iran; 3 Department of Nutrition and Food Science, California State University, Chico, CA USA

**Keywords:** e-learning, Perception, Student, Nutrition, Learning outcomes, COVID-19, Iran

## Abstract

**Background:**

COVID–19 pandemic caused university closures, which created learning challenges for students worldwide. Switching to online educational systems had significant impact on students’ performances. The current study aims to investigate the perception of university students from the Nutrition Science department regarding e-learning in Iran.

**Methods:**

The design of the study is cross-sectional. Data were collected through online surveys from Iranian students from the Nutrition Sciences Department. Stratified random sampling was used to randomly select 955 participants. A self-administered validated questionnaire was used for data collection. Descriptive statistics, Analysis of Variance (ANOVA) and Chi-Square tests were used for analysis of the data.

**Results:**

Results revealed that 67.2% of students didn’t have any former experience of e-learning. About 38.3% had moderate levels of Information Technology (IT) skills. Our results revealed that based on students’ responses, being able to stay at home was one of the most common benefits of e-learning (39.1%). However, the most common challenge that students faced was related to technical problems (39.6%). When compared to e-learning, most students preferred face-to face type of learning. Students believed that this method no only increased their knowledge but also their skills and social competence as compared to e-learning. Only 28% of students rated e-learning as enjoyable. Furthermore, acceptance of online based education was statistically associated with students’ degree level.

**Conclusion:**

In conclusion, students reported both advantages and disadvantages of e-learning but still reported that face-to-face learning is considered the most effective form of learning.

## Background

COVID-19, caused by the SARS-CoV-2, is a contagious disease that spreads rapidly among the human population [1]. The COVID-19 pandemic had disruptive effects on several aspects of human lives such as socio-cultural, socio-economic and educational aspects [2]. In the beginning of the virus outbreak, many educational institutions worldwide were forced to close their campuses in order to protect their students from viral exposures [3]. To continue the education process, universities had no choice but to shift to electronic learning (e-learning) [5]. According to UNESCO monitoring, by end of April 2020 these closures globally affected about 74% of the students worldwide [4]. Thus e-learning became the new face of education, an essential tool to continue education in this crisis [5]. Prior to the COVID-19 pandemic, e-learning was at its early stages at Iranian Universities, offering only a limited number of online programs. However, when the pandemic hit in March 2020, the virtual education system quickly expanded in all educational levels throughout the country [6]. This led to various achievements in technology based tools and online learning systems in which accelerated the development of e-learning [7]. Compared to the academic coursework, web-based instruction allowed learners to continue to access materials which enabled greater flexibility. However, this transition was challenging for those students that had limited access to e-learning and thus it created a lack in learning and social interaction [8].

E-learning is defined as bridging the space between teachers and students through information and communication technology (ITC) to improve the quality of education [9]. It is a type of teaching approach that encompasses learning-related technologies using electronic media and devices [10]. According to literature review, online learning has both advantages and disadvantages [11]. In 2008, Yaghoubi et al. found that Iranian students had a positive perception for e-learning. This perception was influenced by their assessment of e-learning competency, access to the internet and computers, and their evaluation of the higher education system’s shortcomings [12]. Recent studies reported that most Iranian medical students believe that e-learning was an opportunity to overcome academic failure, but it cannot achieve the same effectiveness as face-to-face learning [13–15]. In Malaysia, Almaiah et al. used the technology acceptance model and the innovation and diffusion theory model to identify critical factors influencing the use of e-learning among students. They found that factors such as comparative advantages, observability, flexibility, perceived adaptability, complexity, and enjoyment played a significant role in students’ decision to utilize the e-learning system [16]. Similarly, Salloum et al. discovered that innovation, quality, trust, and knowledge sharing were important factors for achieving acceptance of the e-learning system among students [17]. A cross-sectional study conducted on Italian University Students a year after the COVID-19 pandemic indicated that sociality, stress, quality of life, and coping were important factors that influenced students’ e-learning satisfaction [18].

With this background information, the aim of the present study was to examine the perception of Iranian Nutrition Science students on e-learning during COVID-19 lockdown. It is predicted that the results of this study could be useful in improving e-learning design.

## Methods

The current study was approved by the Institutional Review Board and Ethics Approval Committee of Shahid Beheshti University of Medical Sciences Tehran, Iran (IR.SBMU.SME.REC.1400.101). It adhered to the latest ethical principles of the Declaration of Helsinki [19]. Written informed consent was obtained online from all students before enrollment.

### Study design, population, and sampling

This cross-sectional study was conducted online in twenty medical universities that admitted students for bachelor’s, master’s or doctor of philosophy’s degree in Nutrition Sciences Program between April and November, 2022 in Iran. The study was conducted following the Strengthening the Reporting of Observational studies in Epidemiology (STROBE) guideline [20].

The participants were chosen by a stratified random sampling method. During the sampling process, the population was stratified based on the university three levels of education [bachelor’s (BSc), master’s (MSc) and Doctor of Philosophy (PhD)] as well as gender (male and female). Nutrition students aged 18 years or older were eligible to participate in the study. The inclusion criteria were as follows: giving consent to participate in the study; current Nutrition Science student at Iranian Medical Universities; experience in e-learning during or before the COVID-19 pandemic and having access to the internet. A sample size of 892 students was calculated considering confidence interval of 95%, a response distribution of 50% and a margin of error at 5%. However, the survey link was completely filled by 955 students to account for non-eligibility or non-responders’ rates.

### Study questionnaire and data collection tool

Data collection was done through online forms that were directly sent to eligible students through various social media platforms including WhatsApp, Telegram and Email. Only completed forms were used for final analysis. A self-administered data gathering form included the following sociodemographic details: age, gender, student’s degree level, place of residence, marital status, job status, former experience of e-learning, choice of device used for online learning, and IT skills.

To assess students’ perception of e-learning, a self-administered questionnaire was developed through literature review [13, 15]. This questionnaire was based on the questionnaire used in Maqbool et al.‘s study [13], which was revised according to the Technology Acceptance Model, comprising of two main factors that impact an individual’s inclination to adopt new technology: perceived ease of use and perceived usefulness [21]. The benefits and challenges of e-learning were assessed in terms of the advantages and disadvantages of online education.

Students were asked to select from 10 sets of items that related to benefits and challenges of e-learning. Effectiveness of learning objectives such as clinical skills, social competence and knowledge were measured using a five-point Likert scale by comparing two methods of learning; face-to-face vs. e-learning. The Likert scale ranged from 1 = extremely ineffective to 5 = extremely effective. For the e-learning level of acceptance, Likert scale ranged from 1 = extremely unenjoyable to 5 = extremely enjoyable. The face validity of the questionnaires was determined by 10 students across all academic degree level. The coefficients higher than 1.5 were considered face-valid. The content validity of questionnaire was verified by 12 professors specialized in the fields of nutrition and e-learning using the Lawshe method [22]. In content estimation, students’ perception of e-learning was scaled by content validity ratio (CVR) = 0.95 and content validity index (CVI) = 0.95, respectively.

Data were analyzed using SPSS software (V.23.). The significance level of all tests was targeted at 0.05 (P-value less than 0.05). General characteristics of the students were analyzed by descriptive statistics. Data were expressed as percentages and frequencies or described as mean and standard deviation (SD) for age. Using student’s degree level as the categorical variable, students were placed into three categories: BSc, MSc and PhD. The differences in distributions of all categorical variables were determined using chi-square test, whereas the ANOVA test was used to assess difference in the distribution of age.

## Results

Socio-demographic characteristics of the students are summarized in Table 1. The mean age of students was 23.3 ± 3.52. Out of 955 students participated in our study, 72.1% (n = 689) were female. Overall, distribution of students according to the student’s degree level is as follows: B.Sc. (n = 654, 68.5%), M.Sc. (n = 179, 18.7%) and Ph.D. (n = 122, 12.8%). About 33.6% B.Sc., (n = 220), 20.7% M.Sc. (n = 37), and 45.9% Ph.D. (n = 56) students respectively participated in any e-learning before the COVID-19 pandemic and the results were statistically significant (p-value < 0.001). Mobile phone was the most popular device used among students for e-learning when compared to personal computers, laptops and tablets (50.8%). About 38% of students had moderate levels of IT skills.

**Table 1:** Descriptive statistics of socio-demographic characteristics of the Nutrition science students

	Total (n = 955)	Student’s degree level			P Value^*^
		B.Sc. (n = 654)	M.Sc. (n = 179)	Ph.D. (n = 122)	
Age, Year, Mean (SD)	23.3 ± 3.52	22.1 ± 2.92	23.9 ± 2.24	28.8 ± 2.35	< 0.001
Gender					0.226
Male	266 (27.9)	193 (29.5)	42 (23.5)	31 (25.4)	
Female	689 (72.1)	461 (70.5)	137 (76.5)	91 (74.6)	
Place of residence					0.289
Urban	687 (71.9)	480 (73.4)	121 (67.6)	86 (70.5)	
Rural	268 (28.1)	174 (26.6)	58 (32.4)	36 (29.5)	
Marital status					0.001
Single	821 (86)	579 (88.5)	149 (83.2)	93 (76.2)	
Married	134 (14)	75 (11.5)	30 (16.8)	29 (23.8)	
Job status					< 0.001
Work with school	181 (19)	86 (13.1)	48 (26.8)	75 (61.5)	
Full-time student	774 (81)	568 (86.9)	131 (73.2)	47 (38.5)	
Former experience of e-learning					< 0.001
Yes	313 (32.8)	220 (33.6)	37 (20.7)	56 (45.9)	
No	642 (67.2)	434 (66.4)	142 (79.3)	66 (54.1)	
Choice of device					0.206
PC	119 (12.5)	82 (12.5)	25 (14.0)	12 (9.8)	
Mobile	485 (50.8)	333 (50.9)	85 (47.5)	67 (54.9)	
Laptop	290 (30.4)	203 (31.0)	50 (27.9)	37 (30.3)	
Tablet	61 (6.4)	39 (5.5)	19 (10.6)	6 (4.9)	
IT skills					< 0.001
Low	317 (33.2)	268 (41.0)	27 (15.1)	22 (18.0)	
Moderate	366 (38.3)	222 (33.9)	102 (57.0)	42 (34.4)	
High	272 (28.5)	164 (25.1)	50 (27.9)	58 (47.5)	

BSc, bachelor of science; IT, information technology; MSc, master of science; PC, personal computer; PhD, doctor of philosophy; SD, standard deviation

Values represent the number of subjects (%), except age.

* Chi-square or ANOVA

The overall perception and the categorized responses towards e-learning according to the student’s degree level are shown in Table 2. About 44% of students had positive perception towards e-learning. Compared to MSc and BSc students PhD students had significantly higher positive perception in regards to virtual education (P = 0.007). Most students selected staying at home as one of the benefits of e-learning (39.1%). When students were asked about the challenges of e-learning, majority (39.6%) reported technical problems and 36.1% reported reduced interaction with their professors. Compared to BSc students, MSc and PhD students significantly preferred virtual education for the future learning. (P = 0.014). The perception of doctoral students about the cost effectiveness of e-learning was significantly higher compared to master and undergraduate students (P < 0.001). Undergraduate students perceived home environments to be less suitable for e-learning as compared to post graduate students (P < 0.001). Another challenge of e-learning reported by undergraduate students was the difficulty of adapting to newer e-learning modules and tools (P < 0.001).

**Table 2:** Nutrition science student’s perception on benefits and challenges of e-learning

	Total (n = 955)	Student’s degree level			P Value^*^
		B.Sc. (n = 654)	M.Sc. (n = 179)	Ph.D. (n = 122)	
**Overall perception**					0.007
Positive	422 (44.2)	274 (41.9)	78 (43.6)	70 (57.4)	
Negative	533 (55.8)	380 (58.1)	101 (56.4)	52 (42.6)	
**Benefits of e-learning**					
Being able to stay at home	373 (39.1)	259 (39.6)	72 (40.2)	42 (34.4)	0.526
Adaptable space	369 (38.6)	259 (39.6)	70 (39.1)	40 (32.8)	0.362
Learning on self-paced	310 (32.5)	238 (36.4)	51 (28.5)	39 (32.0)	0.120
Being able to record lectures	269 (28.2)	183 (28.0)	44 (24.6)	42 (34.4)	0.173
Engagement in classes	216 (22.6)	152 (23.2)	37 (20.7)	27 (22.1)	0.760
Long-term cost-effective	229 (24)	133 (20.3)	38 (21.2)	58 (47.5)	< 0.001
Future learning preference	226 (23.7)	137 (20.9)	52 (29.1)	37 (30.3)	0.014
Easy access to online content	174 (18.2)	174 (26.6)	35 (19.6)	28 (23.0)	0.135
The possibility of employment along with education	172 (18)	122 (18.7)	34 (19.0)	16 (13.1)	0.320
Adaptation of different style of learning	142 (14.9)	105 (16.1)	18 (10.1)	19 (15.6)	0.132
**Challenges of e-learning**					
Limited contact with professors	345 (36.1)	251 (38.4)	55 (30.7)	39 (32.0)	0.100
Technical problems	378 (39.6)	255 (39)	72 (40.2)	51 (41.8)	0.828
Limited contact with patients	220 (23.0)	147 (22.5)	35 (19.6)	38 (31.1)	0.053
Home environment not suitable for e-learning	293 (30.7)	240 (36.7)	38 (21.2)	15 (12.3)	< 0.001
Poor self-control	198 (20.7)	146 (22.3)	30 (16.8)	22 (18.0)	0.195
Absence of social contact	272 (28.5)	193 (29.5)	44 (24.6)	35 (28.7)	0.432
Adapting difficulties on implementing newer e-learning modules and tools	262 (27.4)	208 (31.8)	31 (17.3)	23 (18.9)	< 0.001
Struggle with focusing using e-learning	176 (18.4)	129 (19.7)	26 (14.5)	21 (17.2)	0.264
Insecurity of e-learning	194 (20.3)	127 (19.4)	41 (22.9)	26 (21.3)	0.565
More screen-time	191 (20.0)	134 (20.5)	28 (15.6)	29 (23.8)	0.191

BSc, bachelor of science; MSc, master of science; PhD, doctor of philosophy

Values represent the number of subjects (%).

* Chi-square

**Fig. 1:**
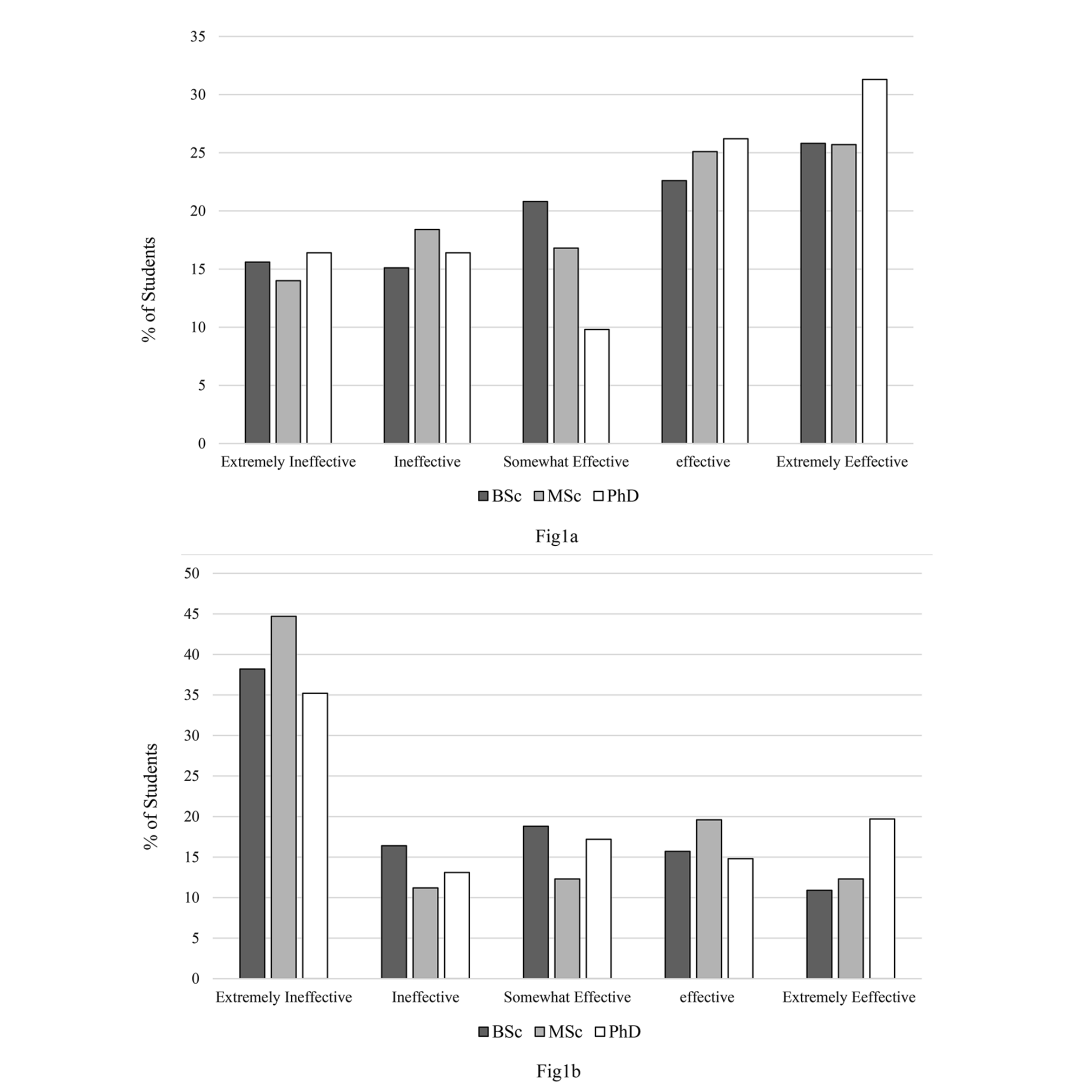
Effectiveness of face-to-face (**a**) and e-learning (**b**) in terms of increasing knowledge according to students degree level

**Fig. 2:**
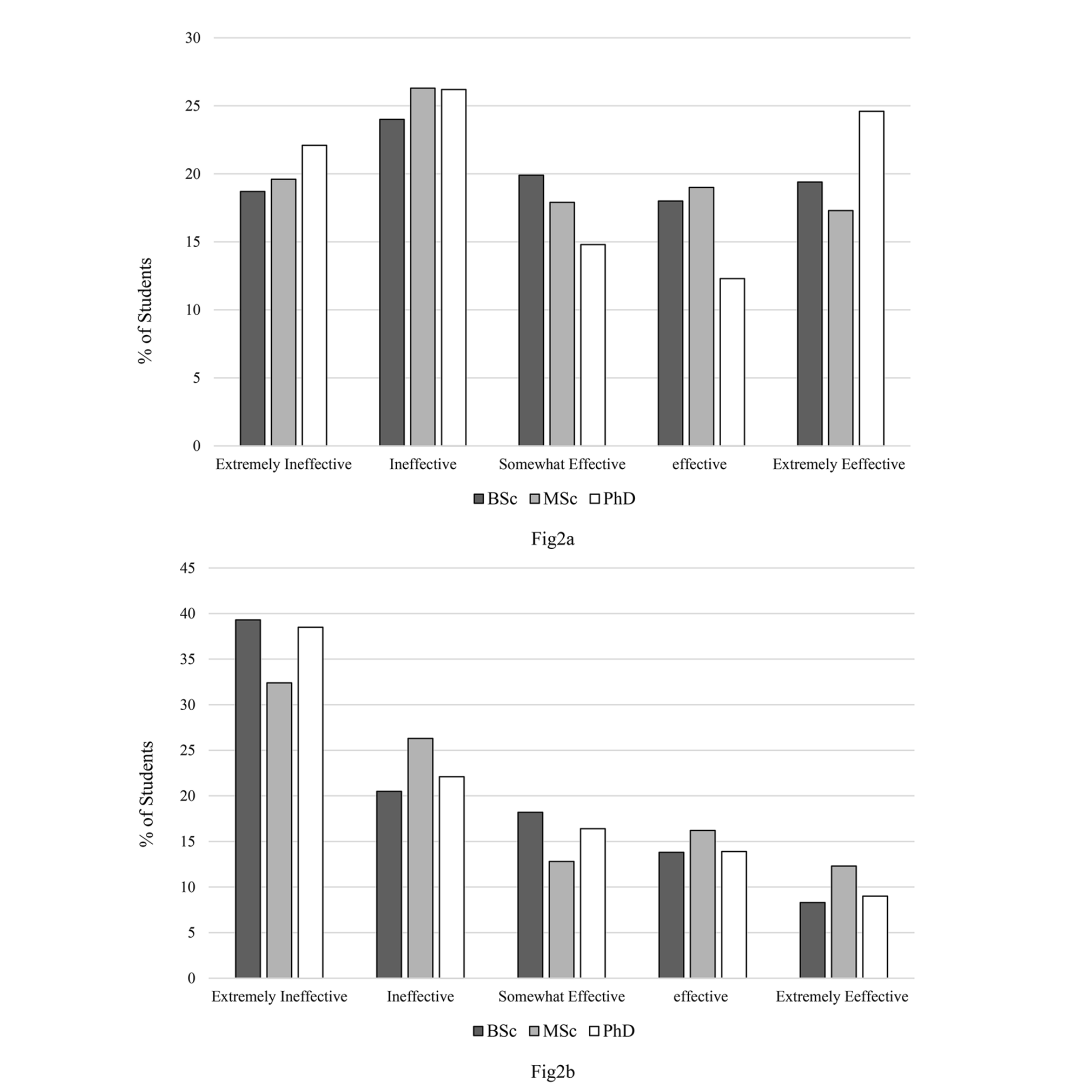
Effectiveness of face-to-face (**a**) and e-learning (**b**) in terms of increasing skill according to students degree level

**Fig. 3:**
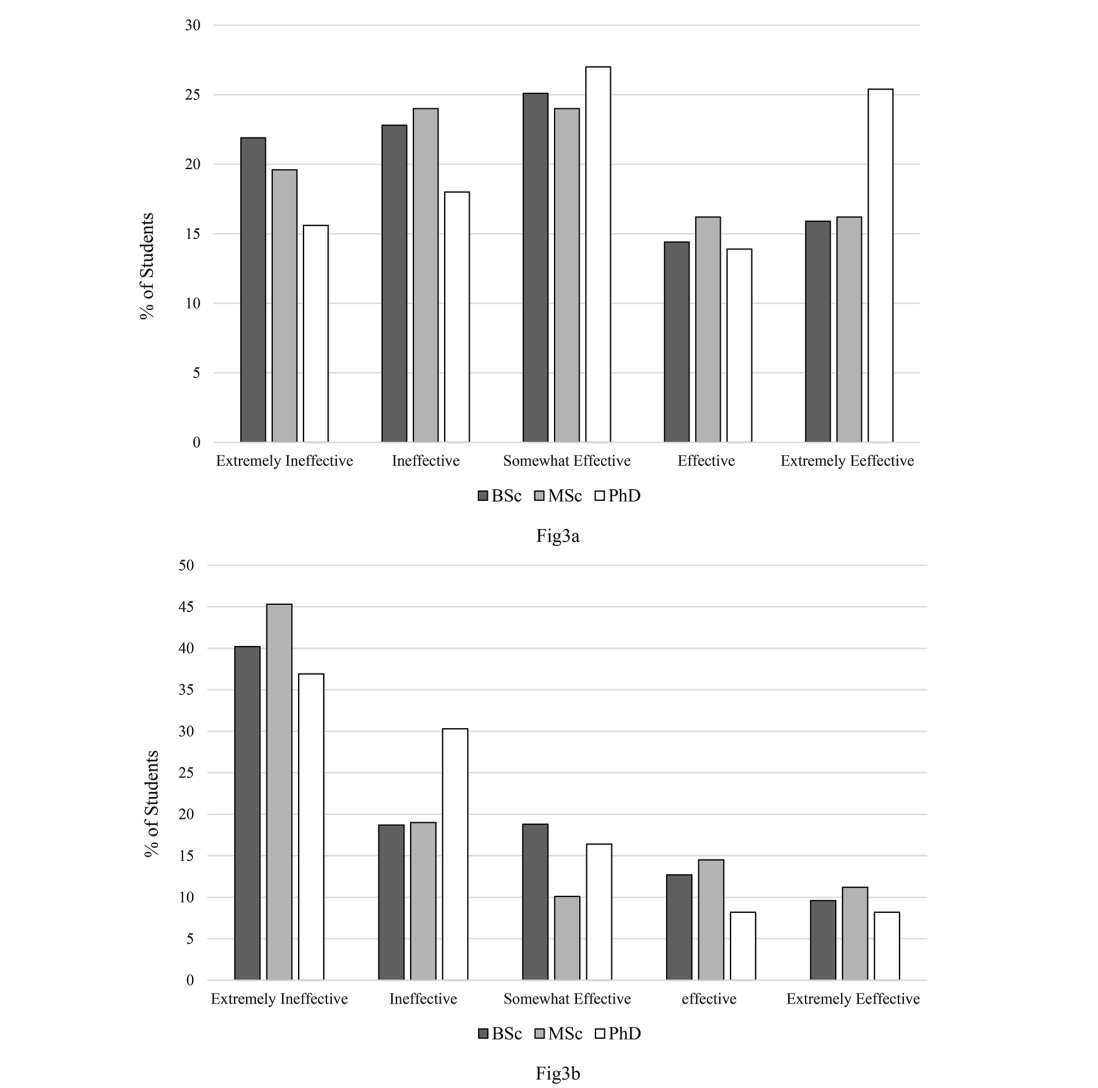
Effectiveness of face-to-face (**a**) and e-learning (**b**) in terms of increasing social competence according to students’ degree level

Effectiveness of face-to-face learning versus e-learning on knowledge, clinical skills, and social competencies are shown in Figs. 1, 2 and 3 respectively. The majority of the students (n = 373, 39.1%) felt that e-learning can be extremely ineffective in terms of knowledge. On the other hand, minority of the students (n = 147, 15.4%) reported face-to-face learning as extremely ineffective.

When asked about skill, about 60% of the students considered the effectiveness of e-learning to be ineffective. However, 44% of students reported the effectiveness of face-to-face learning as ineffective.

About 41% of the students considered the effectiveness of e-learning to be extremely ineffective in terms of social competence and about 21% of students reported the effectiveness of face-to-face learning as extremely ineffective.

**Fig. 4:**
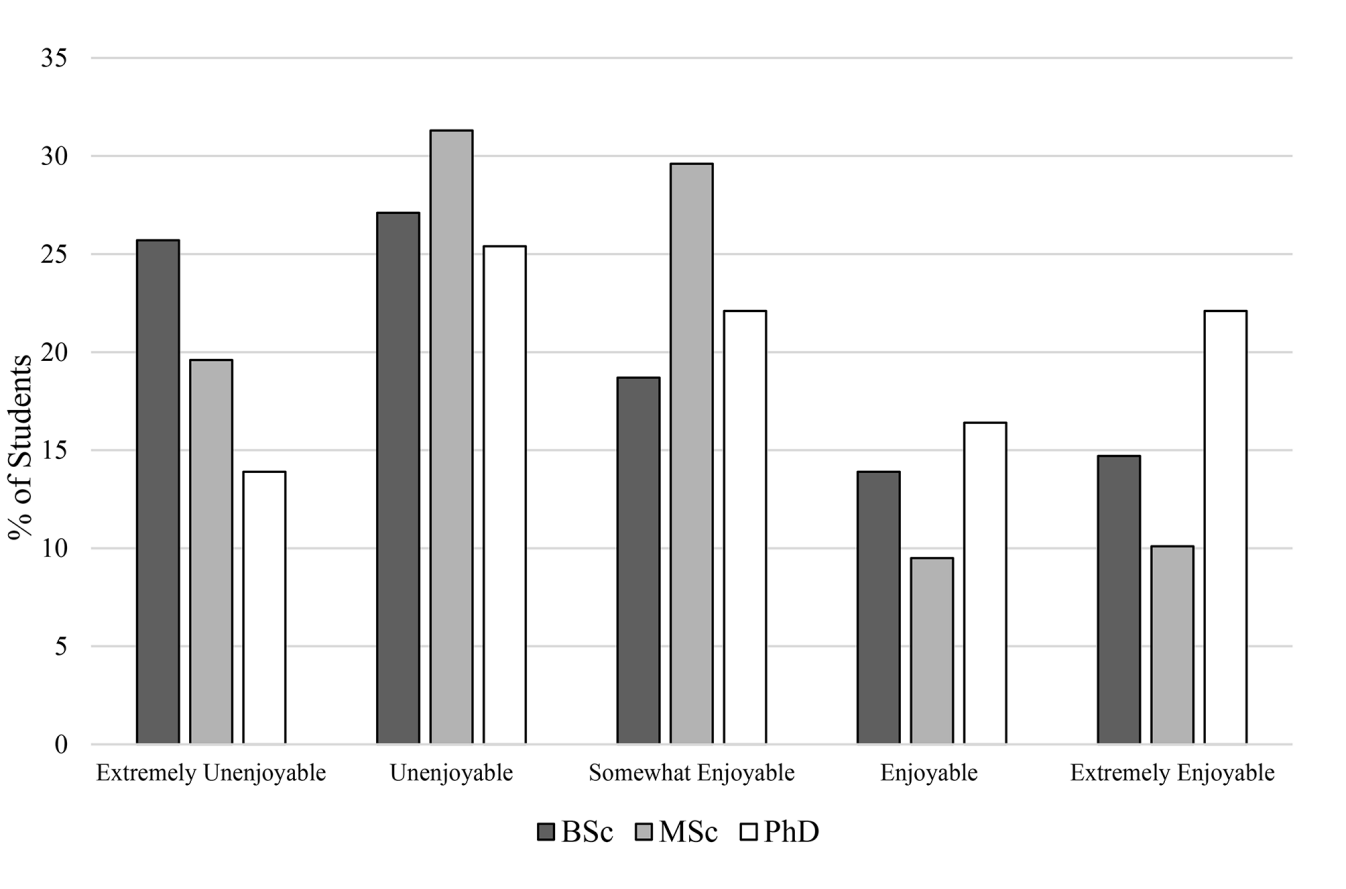
Acceptance of e-learning according to students’ degree level

Acceptance of e-learning according to the students’ degree level is presented in Fig. 4. Out of 955 students, 14.8% (n = 141) found e-learning to be extremely enjoyable and 13.4% as (n = 128) enjoyable while 23% (n = 220,) students did not extremely enjoy e-learning. Acceptance of e-learning was statistically associated with the students’ degree level (P = 0.001) and former experience of e-learning (P = 0.037). However, it was not statistically associated with gender (P = 0.731), device choice (P = 0.638) and IT skills (P = 0.734).

## Discussion

Any information system needs the use of the system by users for its success [23]. One of the main keys to success in e-learning is students’ acceptance of using this method. The current study presents the results of an e-learning assessment by Iranian Nutrition Science students after about two years of COVID-19 pandemic. Our study reported that approximately a little less than half of the students had positive perception towards e-learning. PhD students had significantly more positive perception of e-learning than students of other degrees. The main drawback of e-learning was technical problems which can be mostly due to the network connectivity and internet speed in Iran. Among all students from the three degree levels, being able to stay at home was reported as one of the most advantages of e-learning.

Majority of students responded that switching to e-learning prevented academic failure, however they believed that it cannot be as effective as face-to-face learning. Previous studies reported that majority of university students use mobile devices for e-learning compared to other devices, which is very similar to our study [15, 24–26]. Mobile phones complement e-learning as it can be accessed from anywhere and at any time [22].

In our study, most students had moderate levels of IT skills with no experience in e-learning. These findings were similar to the findings of Maqbool et al. [13].

The primary advantage of e-learning was the ability to stay at home due to flexibility in place and time. In line with our study, Bączek et al. [27] and Maqbool et al. [13] reported the most picked benefits of e-learning was the ability to stay at home. Asif et al. showed that university students in Saudi Arabia had a positive perception towards the online education, with many advantages including flexibility, low cost, self-learning, and convenience [28]. Based on the findings of the study, technical problems including internet connectivity adversely impacted learning. Our data are consistent with Dyrek et al. study. They reported that poor internet connection and quality of classes performed negatively affected e-learning [29]. In a qualitative study conducted by Salahshouri et al. challenges of e-learning in Iran included the structural, equipment and unwillingness to use this educational system, similarly to our study [14]. To overcome this challenge, government bodies in Iran should invest in provision of the infrastructure and allocate enough funds in expanding telecommunication companies for better internet services.

Our findings indicate that when looking at skills, social competence and knowledge, majority of the students perceived that e-learning is less effective than face-to-face. The results of our studies are consistent with other studies when comparing effectiveness of e-learning to face-to-face [13, 30, 31] method. However, in a study conducted on Polish medical students, increase in knowledge was not statistically different when using different learning methods [27]. In a multi-country study, the majority of health care students agreed that e-learning was satisfactory in acquiring knowledge, but not effective in clinical and technical skills [32]. According to the previous report on Polish medical students, e-learning was most effective for development of clinical skills when combined with classroom learning [27]. Saurabh et al. reported that more than half of undergraduate medical students preferred face-to-face learning [33]. Some possible reasons for choosing face-to-face learning method by students are; r interaction between professors and students, better understanding of learning materials, fewer distraction, interactive, less dependency on internet, better collaboration with various departments and more essential for clinical training. Tayem et al. reported medical students had concerns about the clinical skills learning [34].

In our study, acceptance of e-learning was perceived higher in students with higher education level and those had previous experience of e-learning. This could relate to their employment status and cost-effectiveness.

The present study had several limitations. These include the reliance on self-reported data, the omission of a qualitative study, and neglecting psychological distress assessment. Previous studies have reported a correlation between satisfaction with e-learning and stress levels [18, 35], which was not assessed in our study.

In conclusion, the results of this study suggest that there is a significant disparity in the perceived effectiveness of e-learning compared to face-to-face learning across various domains in Iranian students from the Nutrition Science department. The majority of students reported that e-learning was extremely ineffective in terms of knowledge, skill, and social competence. On the other hand, the minority of students found face-to-face learning to be extremely ineffective in these areas. Interestingly, a substantial percentage of students reported e-learning as enjoyable, indicating that enjoyment does not necessarily align with perceived effectiveness. The acceptance of e-learning was found to be influenced by students’ degree level and previous experience, suggesting that familiarity and exposure contribute to its acceptance. However, it is important to note that acceptance of e-learning was not statistically associated with gender, device choice, or IT skills. This implies that these factors may not play a significant role in determining students’ acceptance of e-learning. These findings highlight the need for further research and potential improvements in the design and implementation of e-learning platforms. Overall, this study provides valuable insights into student perceptions and preferences towards e-learning, shedding light on areas for improvement and potential strategies to increase its effectiveness in the future. Moreover, the findings of our research can help policymakers and institutions provide better technology infrastructure for e- learning to succeed.

## Data Availability

Upon a reasonable request, the corresponding author will provide the data that support the findings of this research.
